# Syphilitic Hepatitis Presenting As Cholestatic Liver Injury: A Case Report

**DOI:** 10.7759/cureus.117883

**Published:** 2026-10-13

**Authors:** Mariana Marques, Frederico Duarte, Clara Bacelar, Ricardo Correia de Abreu, José Miguel Sá

**Affiliations:** 1 Internal Medicine, Hospital Vila Franca de Xira, Vila Franca de Xira, PRT; 2 Infectious Diseases, Unidade Local de Saúde de Matosinhos - Hospital Pedro Hispano, Matosinhos, PRT; 3 Critical Care, Unidade Local de Saúde do Alto Minho, Viana do Castelo, PRT

**Keywords:** cholestatic liver injury, secondary syphilis, syphilitic hepatitis, syphilitic rash, treponema pallidum

## Abstract

Syphilis, caused by the spirochete *Treponema pallidum*, is a sexually transmitted infection whose incidence has risen worldwide over the past decade and which may involve virtually any organ, including the liver. Syphilitic hepatitis is an uncommon and frequently overlooked manifestation that characteristically produces a cholestatic pattern of liver injury. We report the case of a 61-year-old man with no relevant medical history and self-reported sexual risk factors, admitted with a 15-day history of bilateral leg edema, fatigue, dry cough, odynophagia, weight loss, and conjunctivitis of the right eye. He denied alcohol consumption and the use of herbal products, dietary supplements, and recreational drugs. Examination revealed erythematous macular lesions on the trunk and limbs sparing the palms and soles, anterior cervical lymphadenopathy, bilateral leg edema, and jaundice, with no palpable hepatomegaly, splenomegaly, or ascites; ophthalmologic assessment excluded uveitis. Laboratory testing showed a cholestatic pattern of liver injury, with alkaline phosphatase and gamma-glutamyl transpeptidase levels of 895 and 981 U/L, respectively, disproportionately elevated compared with alanine aminotransferase and aspartate aminotransferase levels of 103 and 84 U/L, respectively, together with a total bilirubin of 3.16 mg/dL. Serologic tests for hepatitis A, B, C, and E viruses, cytomegalovirus, Epstein-Barr virus, and human immunodeficiency virus were negative, as was a comprehensive autoantibody panel; the iron profile and N-terminal pro-B-type natriuretic peptide were unremarkable. Abdominal ultrasonography and magnetic resonance cholangiopancreatography showed mild hepatomegaly without focal lesions or biliary dilatation. The Venereal Disease Research Laboratory (VDRL) test was reactive at a titer of 1/128, and anti-*Treponema pallidum* IgG was positive. Secondary syphilis with hepatic involvement was diagnosed, and the patient received a single intramuscular dose of 2.4 million IU of benzathine penicillin G. Symptoms resolved, and liver biochemical parameters largely normalized within six months. This case underscores the need to consider syphilis in the differential diagnosis of unexplained cholestatic liver injury, particularly in patients with risk factors for sexually transmitted infections.

## Introduction

Syphilis is a chronic systemic infection caused by the spirochete *Treponema pallidum* subspecies pallidum, transmitted predominantly through sexual contact and vertically during pregnancy [1]. After decades of decline, its incidence has risen steadily: the World Health Organization estimated 8.0 million new infections among adults aged 15 to 49 years in 2022, compared with 7.1 million in 2020 [2]; in high-income countries, the increase has occurred predominantly among men who have sex with men [1].

Untreated infection evolves through primary, secondary, latent, and tertiary stages. The secondary stage reflects hematogenous and lymphatic dissemination of the spirochete. It emerges weeks to months after the primary chancre, which is painless, self-limited, and often unnoticed by the patient [1]. Because dissemination may affect virtually any organ system, syphilis has long been known as the great imitator [1].

Hepatic involvement is among its least recognized manifestations. In the largest systematic review to date, which assembled 144 adult cases published between 1951 and 2017, 88.9% occurred during early syphilis, 4.9% during latent disease and 6.3% during tertiary disease, at a mean age of 40.5 years [3]. Reported frequencies vary widely with case definition, screening intensity, and the population studied, and reached 38% among HIV-infected patients with early syphilis in one cohort [4]. Spirochetes reach the liver via hematogenous dissemination, and histological examination typically shows mixed inflammatory infiltrates in the portal and periportal regions, focal hepatocyte necrosis, and pericholangiolar inflammation. The last accounts for the characteristic cholestatic pattern, in which alkaline phosphatase and gamma-glutamyl transpeptidase, markers of impaired bile flow, rise out of proportion to the aminotransferases [3,5]. Hepatic involvement appears to be more frequent in patients coinfected with HIV [3,4], although no consistent relationship with the degree of immunosuppression has been demonstrated [4,6].

Benzathine penicillin G remains the treatment of choice, and a single intramuscular dose of 2.4 million IU is recommended for early syphilis, including secondary disease; hepatic involvement does not require a modified regimen [6]. Recognition, however, is frequently delayed because presenting symptoms are non-specific, and a cholestatic profile with jaundice more readily suggests biliary obstruction, drug-induced liver injury, or malignancy [7,8]. Delay carries real consequences, since untreated disease may progress to fulminant hepatic failure requiring liver transplantation [9]. Diagnosis relies on serologic testing that combines a non-treponemal test, such as the Venereal Disease Research Laboratory (VDRL) test, whose titer reflects disease activity, with a treponemal-specific assay [10]. No formally validated diagnostic criteria for syphilitic hepatitis exist, and those proposed by Mullick et al. remain the most widely applied [5].

Most published cases of syphilitic hepatitis involve younger patients who are frequently coinfected with HIV and present with the classic palmoplantar rash [3]. We report a case of secondary syphilis presenting predominantly as cholestatic liver injury in an immunocompetent, HIV-negative 61-year-old man whose exanthem spared the palms and soles. These atypical features may contribute to a missed diagnosis. Following a single intramuscular dose of benzathine penicillin G, the patient achieved clinical and serologic resolution, accompanied by near-normalization of liver biochemical parameters.

## Case presentation

We present the case of a 61-year-old man with no significant medical history and no regular medication use, admitted with a 15-day history of bilateral leg edema, fatigue, dry cough, odynophagia, weight loss, and conjunctivitis of the right eye. He denied fever, abdominal pain, photophobia, eye pain, visual loss, vomiting, dyspnea, orthopnea, diarrhea, nasal congestion, chest pain, myalgia, and headache. He reported sexual behaviors associated with an increased risk of sexually transmitted infection. He denied alcohol consumption and the use of herbal products, dietary supplements, and recreational drugs. There was no history of blood transfusion, surgery, or recent travel.

On examination, he was afebrile and hemodynamically stable. Examination revealed erythematous macular lesions on the trunk and limbs, sparing the palms and soles, as well as anterior cervical lymphadenopathy, bilateral leg edema, and jaundice. Cardiac and pulmonary auscultation was normal, and there was no palpable hepatomegaly, splenomegaly, or ascites. Ophthalmologic evaluation ruled out uveitis.

Laboratory evaluation revealed a hemoglobin level of 13.2 g/dL (reference range 13-18 g/dL), leukocytes 6930/µL (4000-11 000/µL) with 4500 neutrophils/µL (1300-8000/µL) and 1700 lymphocytes/µL (1000-4800/µL), platelets 195 000/µL (150 000-400 000/µL), and an erythrocyte sedimentation rate of 20 mm/h (0-20 mm/h). Liver biochemical parameters showed a cholestatic pattern, with alkaline phosphatase 895 U/L (40-150 U/L) and gamma-glutamyl transpeptidase 981 U/L (<55 U/L) disproportionately elevated relative to alanine aminotransferase 103 U/L (<55 U/L) and aspartate aminotransferase 84 U/L (5-34 U/L), together with a total bilirubin of 3.16 mg/dL (0.2-1.2 mg/dL) (Table 1). The R ratio, calculated as (alanine aminotransferase/upper limit of normal)/(alkaline phosphatase/upper limit of normal), was 0.31, consistent with a cholestatic pattern of liver injury (R ≤2). Alkaline phosphatase isoenzymes were not measured; the concomitant elevation of gamma-glutamyl transpeptidase supported a hepatic origin. Prothrombin time, serum electrolytes, glucose, albumin, lactate dehydrogenase, calcium, and renal function were normal. N-terminal pro-B-type natriuretic peptide was 30.2 pg/mL (<125 pg/mL), and lactate was 0.9 mmol/L (<2.0 mmol/L). The iron profile showed serum iron 67 µg/dL (31-144 µg/dL) and transferrin saturation 26% (20-50%), with a mildly raised ferritin of 283 ng/mL (<200 ng/mL).

**Table 1:** Liver biochemistry and syphilis serology at presentation and at four weeks and six months after treatment VDRL: Venereal Disease Research Laboratory

Laboratory parameter	Reference range	At presentation	Four weeks after treatment	Six months after treatment
Alanine aminotransferase (U/L)	<55	103	68	39
Aspartate aminotransferase (U/L)	5-34	84	52	32
Alkaline phosphatase (U/L)	40-150	895	354	150
Gamma-glutamyl transpeptidase (U/L)	<55	981	402	60
Total bilirubin (mg/dL)	0.2-1.2	3.16	1.95	0.5
Albumin (g/dL)	3.5-5.2	3.6	3.6	3.8
International normalized ratio	0.8-1.2	1.1	1	0.9
VDRL	Nonreactive	1/128	1/64	Nonreactive

Testing for HIV was negative. Viral causes of hepatitis were investigated: serologic tests for hepatitis A, B, C, and E viruses, cytomegalovirus, and Epstein-Barr virus were all negative. Antinuclear antibody, anti-smooth muscle antibody, antimitochondrial antibody, antineutrophil cytoplasmic antibody, anti-double-stranded DNA antibody, and anti-liver-kidney microsomal antibody were all negative.

Abdominal ultrasonography and magnetic resonance cholangiopancreatography (MRCP) revealed mild hepatomegaly, with homogeneous hepatic echotexture, no focal hepatic lesions, and no dilatation of the intrahepatic or extrahepatic biliary tree.

Serologic testing for syphilis followed the standard two-test approach [10]. The VDRL test was reactive at a titer of 1/128, and anti-*Treponema pallidum* IgG was positive (index 22.1), confirming the diagnosis of syphilis. Secondary syphilis with hepatic involvement was diagnosed, and the patient was treated with a single intramuscular dose of 2.4 million IU of benzathine penicillin G, in accordance with international guidelines [6].

The patient was followed up in the outpatient clinic, and the exanthem and systemic symptoms resolved. Four weeks after treatment, liver biochemical parameters improved substantially, with alkaline phosphatase decreasing from 895 to 354 U/L, gamma-glutamyl transpeptidase from 981 to 402 U/L, and total bilirubin from 3.16 to 1.95 mg/dL. The VDRL titer also declined to 1/64. At six months, liver biochemical parameters had largely returned to their respective reference ranges, with alkaline phosphatase at the upper limit of normal (150 U/L). The VDRL test became nonreactive at six months and remained nonreactive at 12 months (Table 1). Repeat tests for anti-smooth muscle antibodies and HIV were negative.

## Discussion

Syphilis, often dubbed the great imitator, manifests with a broad spectrum of clinical presentations and may masquerade as hepatitis, an association that frequently goes unrecognized [1]. Hepatic involvement is uncommon and can occur at any stage, as spirochetes invade the liver during hematogenous dissemination. Hepatic involvement appears to be more frequent in patients coinfected with HIV [3,4], although its relationship with the degree of immunosuppression remains uncertain [4].

In the systematic review by Huang et al., which analyzed 144 adult cases, the most frequent clinical features of syphilitic hepatitis were rash (77.9%), fatigue or poor appetite (56.7%), icterus (35.1%), fever (25.8%), weight loss (22.7%) and abdominal pain (21.6%); hepatomegaly was documented in 53.6%, lymphadenopathy in 30.9% and splenomegaly in 14.4% [3]. A cholestatic pattern of liver enzyme elevation predominates [3,11], and in an early series of 175 patients with syphilis, hepatitis was confirmed histologically in 17 [12]. The diagnosis of syphilitic hepatitis remains clinical and is established according to the criteria proposed by Mullick et al. [5], all four of which were met in the present case.

First, liver enzyme abnormalities followed a predominantly cholestatic pattern, with disproportionate elevation of alkaline phosphatase (895 U/L) and gamma-glutamyl transpeptidase (981 U/L) compared with relatively mild elevations in aminotransferase levels (alanine aminotransferase 103 U/L, aspartate aminotransferase 84 U/L), together with a total bilirubin of 3.16 mg/dL and an R ratio of 0.31. The parallel elevation and subsequent decline of gamma-glutamyl transpeptidase supported a hepatic origin of the alkaline phosphatase. Second, serologic evidence of active infection was documented by a reactive VDRL test with a titer of 1/128 and a positive anti-*Treponema pallidum* IgG in a patient with concurrent cutaneous manifestations of secondary syphilis.

Third, alternative etiologies were investigated and considered unlikely. Viral hepatitis was considered unlikely based on negative serologic test results for hepatitis A, B, C, and E viruses, as well as for cytomegalovirus and Epstein-Barr virus, and HIV testing was negative both at presentation and at follow-up. Autoimmune liver disease was considered unlikely given negative antinuclear, anti-smooth muscle, antimitochondrial, antineutrophil cytoplasmic, anti-double-stranded DNA, and anti-liver-kidney microsomal antibodies. Toxic and drug-induced liver injury was considered unlikely based on the patient's reported absence of alcohol consumption and use of herbal products, dietary supplements, or recreational drugs.

Iron studies did not support hereditary hemochromatosis, with a serum iron of 67 µg/dL and a transferrin saturation index of 26%; the mildly elevated ferritin of 283 ng/mL was interpreted as an acute-phase response rather than evidence of iron overload. Congestive hepatopathy secondary to heart failure was excluded by an N-terminal pro-B-type natriuretic peptide of 30.2 pg/mL, a normal cardiopulmonary examination, and the absence of dyspnea or orthopnea. Sepsis-associated cholestasis was considered improbable in an afebrile, hemodynamically stable patient with a normal leukocyte count, an erythrocyte sedimentation rate at the upper limit of normal, and a lactate of 0.9 mmol/L. Finally, abdominal ultrasonography and MRCP demonstrated no biliary obstruction and no features suggestive of malignancy.

Fourth, biochemical and serologic improvement followed antitreponemal therapy. Four weeks after a single dose of benzathine penicillin G, alkaline phosphatase and gamma-glutamyl transpeptidase levels had each decreased by approximately 60%, and the VDRL titer had declined from 1/128 to 1/64. By six months, liver biochemical parameters had largely returned to their respective reference ranges, with alkaline phosphatase at the upper limit of normal. The VDRL test became nonreactive at six months and remained nonreactive at the 12-month follow-up (Table 1). An adequate serologic response after treatment of early syphilis is defined as a fourfold or greater decline in non-treponemal titer within six months, and a nonreactive test is considered the best confirmation of cure; in this patient, the VDRL remained nonreactive at the 12-month follow-up recommended by current guidelines [6], confirming a sustained serologic response.

Because all four criteria were met and the treatment response was clear, we did not pursue liver biopsy. The classic histological description of hepatic syphilis, reported by Hahn in a large autopsy and biopsy series, comprises portal inflammatory infiltrates with focal hepatocyte necrosis and pericholangiolar involvement [13]; histological confirmation retains a role in atypical presentations or when the biochemical response to therapy is incomplete [8].

Several features of this case merit emphasis. The exanthem spared the palms and soles, eliminating a characteristic clinical clue to secondary syphilis. Furthermore, the patient was neither immunosuppressed nor HIV-infected, making syphilitic hepatitis less likely to be suspected [4]. At 61 years, he was also two decades older than the mean age reported in the largest published series [3]. Hepatomegaly was not detectable on palpation and was identified only on imaging, which illustrates why cross-sectional or ultrasonographic assessment should not be omitted when the physical examination is unremarkable. Taken together, these features illustrate a clinical scenario in which syphilitic hepatitis may be overlooked, highlighting the importance of maintaining a low threshold for syphilis testing.

The temporal sequence further supports the diagnosis of secondary rather than latent or tertiary disease. The patient presented with a 15-day history of constitutional and systemic symptoms, including fatigue, weight loss, odynophagia, dry cough, unilateral conjunctivitis, and bilateral leg edema. These findings are consistent with secondary syphilis, in which disseminated manifestations typically emerge weeks to months after the primary lesion. The absence of a recalled primary lesion does not argue against the diagnosis, since the initial ulceration is painless, self-limited, and frequently unrecognized by the patient. The concurrent presence at admission of a truncal and limb macular exanthem, anterior cervical lymphadenopathy, and a high non-treponemal titer is characteristic of the secondary stage, as VDRL titers parallel the bacterial burden of active dissemination. Cholestatic liver injury was documented at the same time point as the systemic manifestations, indicating hepatic involvement within the same disseminated episode rather than a sequela of long-standing untreated infection.

The close temporal relationship between antitreponemal therapy and both clinical and biochemical recovery, in the absence of any alternative intervention, supports a causal rather than coincidental association between syphilitic infection and the liver injury observed. However, without histological confirmation, causality cannot be established with certainty.

This report has limitations. Liver biopsy was not performed, so histological confirmation is lacking, and dark-field microscopy and polymerase chain reaction testing of the skin lesions were not carried out. The diagnosis therefore rests on serologic findings, the exclusion of alternative causes, and the response to treatment, as is the case in most published reports of syphilitic hepatitis. Without histology, alternative causes of liver injury cannot be fully excluded. The imaging findings come from the formal radiology reports: both studies were requested during admission but performed at a partner institution, and only the written reports, not the images themselves, were released to the admitting hospital. Therefore, no imaging figure could be included.

## Conclusions

Syphilitic hepatitis is a rare but treatable cause of cholestatic liver injury that is easily overlooked, particularly when the characteristic palmoplantar rash is absent and the patient is neither young nor immunosuppressed. In this immunocompetent, HIV-negative 61-year-old man, disproportionate elevations in alkaline phosphatase and gamma-glutamyl transpeptidase levels, jaundice, a macular exanthem, and self-reported sexual risk factors prompted serologic testing for syphilis and led to the diagnosis. A single intramuscular dose of benzathine penicillin G was followed by clinical and serologic resolution, with near-normalization of liver biochemical parameters within six months. Although the diagnosis rests on clinical, biochemical, and serologic criteria rather than histological confirmation or direct detection of *Treponema pallidum* in hepatic tissue, the overall picture strongly supports syphilitic hepatitis.

Because untreated syphilitic hepatitis may progress to fulminant hepatic failure and effective treatment is inexpensive and generally well tolerated, serologic testing for syphilis should be considered early in the evaluation of unexplained cholestatic liver injury, particularly in patients with risk factors for sexually transmitted infections. Recognizing the hepatic presentation also allows treatment before progression to tertiary disease and enables partner notification, with benefits that extend beyond the individual patient.
